# Metabonomic Analysis of Silkworm Midgut Reveals Differences between the Physiological Effects of an Artificial and Mulberry Leaf Diet

**DOI:** 10.3390/insects14040347

**Published:** 2023-03-31

**Authors:** Juan Li, Jing Deng, Xuan Deng, Lianlian Liu, Xingfu Zha

**Affiliations:** 1 State Key Laboratory of Silkworm Genome Biology, Institute of Sericulture and Systems Biology, College of Sericulture, Textile and Biomass Sciences, Southwest University, Chongqing 400715, China; 2 School of Life Sciences, Southwest University, Chongqing 400715, China

**Keywords:** artificial diet, silkworm, midgut, mulberry leaf, metabolomics

## Abstract

**Simple Summary:**

A metabolomic analysis was carried out on the midguts of silkworms fed either on an artificial diet or fresh mulberry leaves, to examine the effects of different dietary constituents on silkworm physiology. Significant effects were observed on protein synthesis, immunity and disease resistance, silk protein quality, and silkworm size.

**Abstract:**

*Bombyx mori* is a model lepidopteran insect of great economic value. Mulberry leaves are its only natural food source. The development of artificial diets can not only resolve the seasonal shortage of mulberry leaves but also enable changes to be made to the feed composition according to need. Metabolomic differences between the midguts of male and female silkworms fed either on fresh mulberry leaves or an artificial diet were studied using liquid chromatography–mass spectrography (LC-MS/MS) analysis. A total of 758 differential metabolites were identified. Our analysis showed that they were mainly involved in disease resistance and immunity, silk quality, and silkworm growth and development. These experimental results provide insights into the formulation of optimized artificial feed for silkworms.

## 1. Introduction

The silkworm (*Bombyx mori*) is an important cash crop insect and a model lepidopteran species. Wild silkworms were domesticated more than 5000 years ago [1]. They have high economic and research value, and their evolution, survival, and reproduction depend entirely on human agency [2]. Silkworms are a typical monophagous insect and eat only mulberry leaves (ML) [3] to obtain the nutrients they require. However, ML are only available seasonally, and this has become a major problem in silkworm rearing because the period of availability is short and they are difficult to preserve. To address this problem, many researchers have resorted to an artificial silkworm diet (AD). As early as the 1970s, Japanese researchers used semi-synthetic diets to feed silkworm larvae and achieved normal larval growth with low mortality [4,5]. In the 1980s, some scholars studied the relationship between an AD and the levels of diapause hormone [6]. Chinese scientists also conducted many studies on the effects of AD on silkworm growth during the 1980s [7]. An AD that can be easily stored can not only solve the problem of the seasonal availability of ML but also optimize various silkworm traits and silk production performance through changes in their nutritional composition [8,9,10].

The midgut plays an important role in a silkworm’s development and metabolism and is the main site of digestion, absorption, and innate immunity during their growth and development [11]. Differing nutrient compositions of an AD greatly affect the midgut metabolites of silkworms, affecting their growth, development, and silk yield. For example, silk protein has a unique amino acid composition [12] in which glycine (Gly) plays an important role in regulating silk protein synthesis [13,14,15]. Amino acid metabolites in the silkworm midgut can indirectly reflect the rate of silk protein production, and the silk yield can be effectively increased by adding Gly to an AD.

Although artificial feed brings great convenience to sericulture, silkworms reared on artificial feed alone are inferior to those reared on ML in silk performance, larval survival rate, disease resistance, and other aspects in all instars [3,16]. Some scholars have observed differences in the proteome of silkworm pupae under artificial feeding regimes, compared with those fed on ML. Differences in lipid transport and metabolism in silkworm pupae under different nutritional regimes may affect hormone secretion [17]. The levels of amino acids, carbohydrates, and lipids decreased significantly in the feces of silkworms fed on an AD, while the levels of organic acids, such as urea and citric acid, increased significantly [18]. As ADs continue to improve, understanding the nutritional composition of ML is key to their development. The silkworm midgut is the main site of food digestion and absorption. Analyses of midgut metabolites are helpful in understanding the nutritional composition and functions of food, and as a way to provide a useful basis for further optimizing ADs.

## 2. Materials and Methods

### 2.1. Preparation of Artificial Feed and Mulberry Leaves

The artificial diet consisted of mulberry leaf powder, soybean meal, corn meal, vitamin B complex, carrageenan, sorbic acid, gallic acid, ascorbic acid, and mixed inorganic salts. The ingredients were mixed evenly, and ddH_2_O was added at 1.5 times the mass ratio, stirred evenly, and then put into stainless steel lunch boxes. After 20 min at 121 °C, the mixture was removed and cooled to room temperature, and then stored in a refrigerator at 4 °C. The fresh mulberry leaves were provided by the Mulberry Garden (Southwest University, Chongqing, China).

### 2.2. Silkworms Rearing

The silkworms (*Dazao*) were provided by the State Key Laboratory of Silkworm Genome Biology (Southwest University, Chongqing, China), and kept at an ambient temperature of about 25 °C and ambient humidity of 75%.

### 2.3. Silkworm Feeding Trial

Male and female individuals were fed separately until the first day of the fifth instar, and the middle intestinal tissue was taken on ice after the fifth instar after three days. Four experimental dietary treatment groups were established by sex and feeding method: males fed on AD (group MG_AD); males fed on a pure ML diet (group MG_ML); females fed on AD (group FG_AD); and females fed on a pure ML diet (group FG_ML). The feeding trial started at the 1st day of the fifth instar and finished on the 3rd day of the fifth instar.

### 2.4. Sample Preparation and Collection

When the silkworms had grown to the third day of the fifth instar, the midgut tissue of eight replicate silkworms from each feeding trial group was removed. Excess tissue was removed from the sample material in PBS, washed three times, and the excess water was then blotted out on a pressurized filter paper. Samples were then frozen in liquid nitrogen in a 1.5 mL RNase-free centrifuge tube and placed in a refrigerator at −80 °C.

### 2.5. Metabolite Extraction

Samples of 100 mg were taken from the pooled midgut samples from each feeding group, mixed with liquid nitrogen, and the homogenate was then resuspended with prechilled 80% methanol in a well vortex. The samples were incubated on ice for 5 min and were then centrifuged for 20 min at 15,000× *g* and 4 °C. Some of the supernatant was diluted to a final concentration containing 53% methanol using LC-MS grade water. The samples were subsequently transferred to fresh Eppendorf tubes and then were centrifuged for 20 min at 15,000× *g* and 4 °C. Finally, the supernatant was injected into a liquid chromatograph with tandem mass spectrometry (LC-MS/MS) system for analysis (QTRAP ^®®^ 6500+, Sciex, Framingham, MA, USA). Liquid chromatography–mass spectrometry is a new metabolome detection technology with liquid chromatography as a separation system and mass spectrometry as a detection system. A blank sample containing 53% methanol solution made up with water was used to replace the pretreatment process of the experimental samples with that of experimental sample, and a quality control (QC) sample was prepared containing equal volumes of each experimental sample and mixed evenly.

### 2.6. LC-MS/MS Analysis

The prepared samples were subjected to targeted metabolomics study using LC-MS technology based on the highly sensitive SCIEX QTRAP ^®®^ 6500 + mass spectrometry platform. A multi-response monitoring model (MRM), based on the Novogene database (NovoDB, Nuohe Zhiyuan Company, Beijing, China), was used to analyze the experimental samples. Compounds were quantified using product ion (Q3) and relatively qualitatively analyzed using parent ion (Q1). The Q1, Q3, retention time (RT), declustering potential (DP), and collision energy (CE) were used for metabolite identification. The SCIEX OSV1.4 software was used to open the off-machine mass spectrometry file to integrate and correct the chromatographic peaks, which were screened according to a minimum peak height of 500, signal-to-noise ratio of 5, and number of smoothing points of 1. The area under each chromatographic peak represents the relative amount of the corresponding substance. Finally, the area integral data of all the chromatographic peaks were derived to obtain qualitative and quantitative metabolite results.

### 2.7. Processing and Statistical Analysis of Metabolomics Data

The metabolites identified were annotated using the KEGG (https://www.genome.jp/kegg/pathway.html (accessed on 3 June 2021)), HMDB (https://hmdb.ca/metabolites (accessed on 7 June 2021)), and LIPIDMaps (http://www.lipidmaps.org/ (accessed on 20 June 2021)) databases. The data were transformed prior to multivariate statistical analysis using the metabolomics data processing software metaX, and then subjected to principal component analysis (PCA) and partial least squares discriminant analysis (PLS-DA) to obtain the variable importance of projection (VIP) values for each metabolite. In the univariate analyses, the statistical significance (*p*-value) was calculated for each metabolite between the two diet groups using the *t*-test, and the fold change (FC) value was calculated for the differences in the metabolites between the two groups. All of the statistical analyses were performed using the programs in R (https://www.r-project.org/ (accessed on 12 July 2021)).

## 3. Results

### 3.1. Phenotypic Observations and Metabolite Analysis

The experimental diets comprised four groups by sex and feeding method, as described in Section 2.3, and their midgut tissues were excised for metabolomic analysis. All of the groups were reared under the same temperature and humidity conditions, as described in Section 2.2. To study the effects of an AD on silkworms, we examined the phenotypes of silkworms fed on the AD and ML until the third day of the fifth instar. We found that the silkworms fed on the AD grew slightly larger than those fed on ML (Supplementary Figure S1). This is consistent with what we observed in our previous articles. The reason for this phenomenon may be that silkworms reared on an artificial diet are longer than those that are mulberry-leaf-reared at larval stage. Although the growth rate of silkworms fed an AD is slower, they also eat more food during the longer period spent in each instar, taking more time to grow to the third day of the fifth instar, and so they have a larger body size, at the same stage, than silkworms fed on ML.

**Figure 1:**
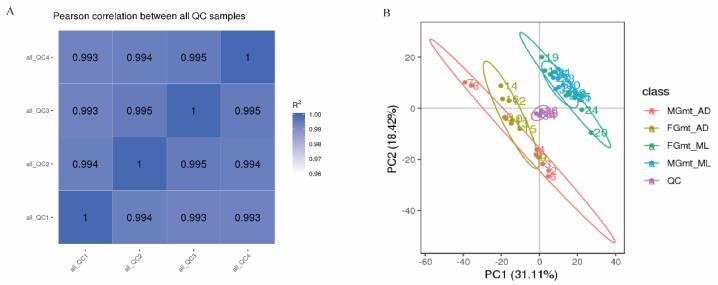
Correlation analysis and PCA. The correlation analysis is shown in (**A**), the distance heat map, showing the Pearson correlation coefficient values between samples. The principal component analysis based on eight replications of MG_AD, FG_AD, FG_ML, and MG_ML is shown in (**B**).

Our metabonomic analysis identified 758 metabolites in the various feeding regimes in this study. To assess the biological impacts of the different metabolites in each group, we calculated the Pearson correlation coefficients for the QC samples (Figure 1A), which showed that the samples were well related. In addition, the total ion current (TIC) curves of the different QC samples, measured and analyzed using mass spectrometry, showed high overlap (Supplementary Figure S2); that is, the retention time and peak intensity were consistent, indicating that the signals were stable and the detection results reliable when the same sample was measured at different times, allowing the next stage of analysis to be performed with confidence.

Principal component analysis (PCA) was used to evaluate the correlation between the midgut metabolites in the two groups, and to detect the overall intergroup metabolic differences and the within-group variability. Comparisons were made between the groups fed on ML and the AD, including female midgut (FG) and male midgut (MG) tissues. The peaks extracted from the eight independent replicate experimental samples and the QC samples were first processed using univariate (UV) scaling and then PCA analysis (Figure 1B). After dimensionality reduction, the difference in the contribution of the abscissa PC1 to the between-group samples was 31.85%, and was mainly driven by the different diets. The contribution rate of ordinate PC2 to the difference between the groups was 18.94%, and was driven by the different genders. FG_ML and MG_ML were incompletely separated, as were the MG_AD and FG_AD groups, but the AD and ML treatment groups were completely separated, indicating significant differences in the metabolic profiles.

### 3.2. Differential Metabolite Screening

**Figure 2:**
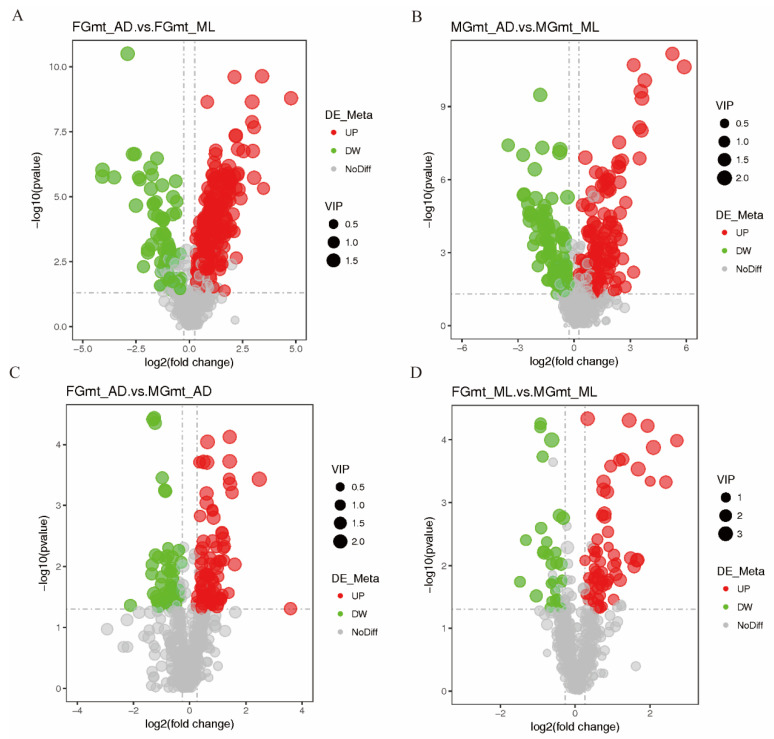
Volcano map of metabolites from silkworms of different genders fed on different diets. (**A**) FG_mt__AD. vs. FG_mt__ML; (**B**) MG_mt__AD. vs. MG_mt__ML; (**C**) FG_mt__AD. vs. MG_mt__AD; (**D**) FG_mt__ML. vs. MG_mt__ML. The horizontal coordinate indicates the change in the multiplicity of differences in metabolites across subgroups (log2FC) and the vertical coordinate indicates the level of significance of the differences (−log10(*p*-value)). Significantly up-regulated metabolites are indicated by red dots and significantly down-regulated metabolites by green dots, with the size of the dots representing the VIP values.

Screening of the differential metabolites was based on the PLS-DA model (Supplementary Figure S3), combined with univariate *p*-values and differential fold change (FC). Under the conditions of VIP > 1.0, FC > 1.2, or FC < 0.833, and *p*-value < 0.05, 758 metabolites were identified (Supplementary Table S1). A total of 280 differential metabolites were screened from MG_AD/MG_ML, of which 183 were up-regulated and 97 were down-regulated. A total of 318 differential metabolites were screened from FG_AD/FG_ML, of which 245 were up-regulated and 73 were down-regulated. A total of 139 differential metabolites were obtained by FG_AD/MG_AD screening, of which 90 were up-regulated and 49 were down-regulated. A total of 95 differential metabolites were screened from FG_ML/MG_ML screening, of which 65 were up-regulated and 30 were down-regulated (Table 1 and Supplementary Table S1, Figure 2).

There were fewer differential metabolites between FG_AD vs. MG_AD and FG_ML vs. MG_ML than between MG_AD vs. MG_ML and FG_AD vs. FG_ML. The differential metabolites between FG_AD vs. MG_AD and FG_ML vs. MG_ML were mainly amino acids and their derivatives, nucleotides and their derivatives, organic acids and their derivatives, phospholipids, etc. The differences in these metabolites were mainly due to the different sexes of the silkworms. There were significantly more different metabolites in silkworms fed on AD than those fed on ML. The main metabolite differences between FG_AD and FG_ML were in amino acids and their derivatives, nucleotides and their derivatives, organic acids and their derivatives, and fatty acyl groups. The significantly down-regulated metabolites were 18-hydroxycorticosterone, 20-carboxyleucotriene B4, 2-methylbutyryl carnitine, and the significantly up-regulated metabolites were Nε-(1-carboxymethyl)-L-lysine, methionine sulfoxide, 2,6-diaminoheptadecic acid, and other substances. The main differential metabolites between MG_AD vs. MG_ML were amino acids and their derivatives, nucleotides and their derivatives, organic acids and their derivatives, carbohydrates and their derivatives, phospholipids, and fatty acyl. The three most significantly up-regulated and down-regulated metabolites were the same as for FG_AD vs. FG_ML (Table 2), indicating that the differences in the major metabolites were caused by the different diets, with gender differences having less of an effect. The types of differential metabolites observed may reflect a series of physiological processes. For example, protein anabolism and differential gene expression in the silkworm midgut will be different under different nutritional regimes, which will affect the development of silkworm tissues and organs, and may affect the size of silkworms and silk yield to some extent. These result provide insights into the formulation of the optimal AD for silkworm feeding.

### 3.3. Differential Metabolite Correlation and Functional Classification

In order to further understand the relationships between the differential metabolites and nutrient metabolism in the different diet treatment groups, we plotted a metabolite clustering heat map and performed a KEGG analysis on the differential metabolites detected.

**Figure 3:**
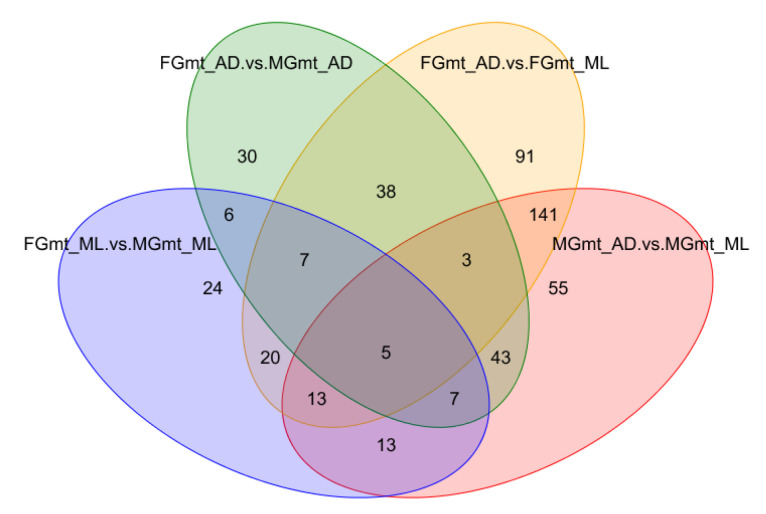
Venn diagram of the differential metabolites. The diagram illustrates the unique and common differential metabolites in the FG_AD vs. FG_ML, MG_AD vs. MG_ML, MG_AD vs. FG_AD and MG_ML vs. FG_ML groups, based on a statistical significance cut-off of *p*-adj < 0.05.

The differential metabolites from the different diet groups were first plotted in a Venn diagram (Figure 3) to visualize the overlaps and unique differential metabolites between the groups. The figure shows 24, 30, 91, and 55 individual differential metabolites in the four diet treatments. The five differential metabolites in common were Xanthosine, 2-Phenylbutyric acid, L-methionine sulfone, D-Homocysteine, and 1-(4-Methoxyphenyl)-2-propanone. The different metabolites in the four experimental groups may play important roles in silkworm nutrient absorption and metabolism.

**Figure 4:**
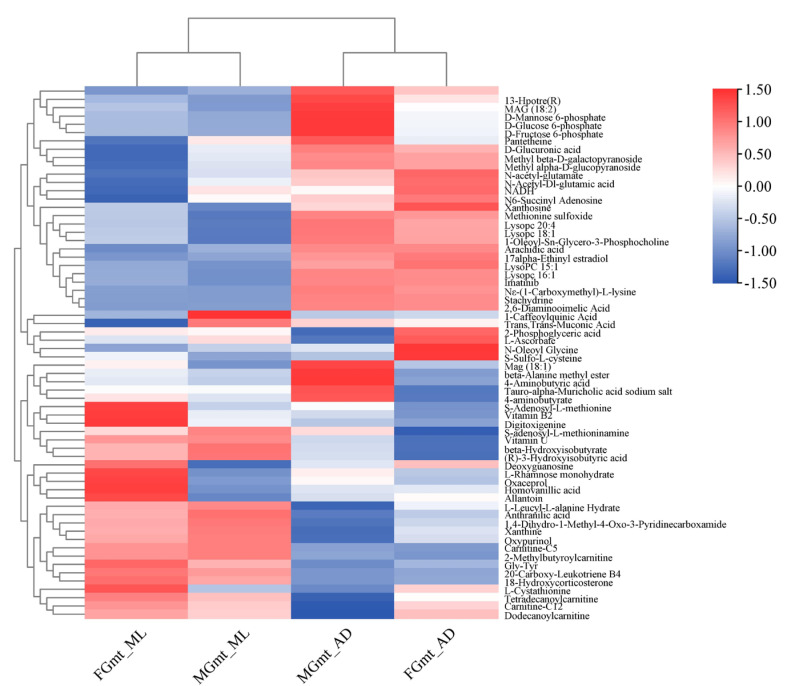
Total differential metabolite cluster heat map. The vertical axis shows the clustering of samples, the horizontal shows the clustering of metabolites; the shorter the cluster branch, the higher the similarity. The depth of the red color represents the relative level of the up-regulated metabolites, and blue represents down-regulated metabolic pathways.

We screened out the top ten metabolites that were significantly up-regulated and down-regulated in each group comparison for heat map analysis (Figure 4) to observe abundance patterns. The differential metabolites of FG_ML and MG_ML were closely related to each other, as were those of FG_AD and MG_AD. The differences in differential metabolites were also obvious in the different feeding regimes in the same sex, indicating that diet had a greater effect on the differential metabolites than sex.

**Figure 5:**
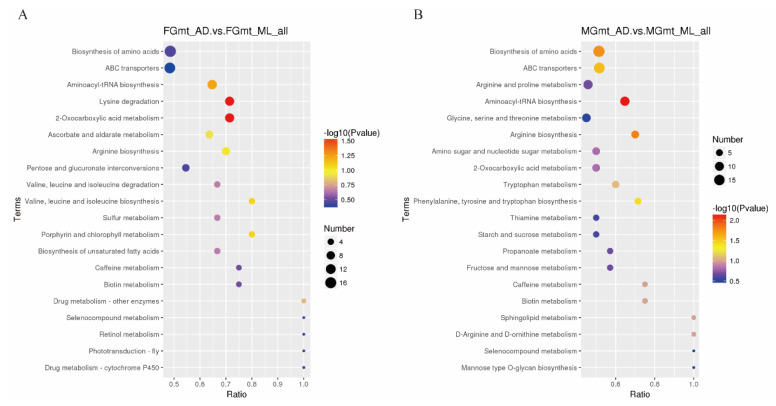
KEGG enrichment. (**A**) FG_AD vs. FG_ML; (**B**) MG_AD vs. MG_ML. Only the top 20 differentially enriched metabolite enrichment pathways are shown. The horizontal coordinate represents x/y (the number of differential metabolites in the corresponding metabolic pathway/the number of total metabolites identified in that pathway), with larger values indicating greater enrichment of differential metabolites in that pathway. The color of the dot represents the *p*-value of the hypergeometric test; the smaller the value, the more reliable and statistically significant the test is. The size of the dot represents the number of differential metabolites in the corresponding pathway; the larger the dot, the more differential metabolites are present within that pathway.

To explore the relationship between the 758 differential metabolites and the function of the silkworm organismal function, the KEGG database [19] was used to annotate the functions of the differential metabolites (Figure 5). In the FG_AD/FG_ML group, 318 metabolites were enriched in 42 pathways. Among these, the greatest number of enriched metabolites were in the amino acid biosynthesis pathway, ABC transporter signaling pathway, aminoacyl-tRNA synthesis pathway, lysine digestion pathway, and 2-oxy-carboxylic acid metabolic pathway. In the MG_AD/MG_ML group, 280 differential metabolites were enriched in 48 pathways. Interestingly, both MG_AD/MG_ML and FG_AD/FG_ML showed more enriched metabolites in the first two pathways, but MG_AD/MG_ML also showed more enriched metabolites in the arginine and proline metabolic pathway, and the glycine, serine, and threonine metabolic pathway. The level of metabolite differences between these two pathways was influenced by the sex of a silkworm, with males being more significantly enriched in both pathways and showing effects in more amino acid metabolic pathways than females.

### 3.4. Proteome and Metabolome Association Analysis

Proteomics studies can reveal the changes that are taking place in organisms, while metabolomics can tell us the changes that have taken place in organisms. We combined proteomic and metabolomic analyses and mapped both the differential proteins and metabolites to the KEGG pathway database to determine the signal transduction pathways and biochemical pathways involving both the proteomic and metabolomic analyses. Pathway information related to the differential proteins (Supplementary Table S2) and metabolites was pooled for the AD and ML groups of female silkworms (Table 3), and was mainly related to metabolic pathways such as amino acid metabolism, pyrimidine metabolism, and pyruvate metabolism. The common pathway in male silkworms was also mainly related to the metabolism and biosynthesis of amino acids (Table 4). Therefore, whether at the proteome or metabolome level, the process of amino acid metabolism and synthesis was more affected in silkworms fed on the AD than those fed on ML.

## 4. Discussion

The metabolites found in the midguts of silkworms are closely related to an individual silkworm’s disease resistance [20], size, and silk protein content [21]. Assessing the metabolites and metabolic pathways in the midgut is helpful in improving the various traits of silkworms. Knowing the metabolites present in the midguts of silkworms can also indirectly reveal the effects of different silkworm nutritional regimes. However, little is known about the differences in midgut metabolomes between silkworms fed an AD and ML.

The ABC transporter belongs to a family of membrane proteins [22], and animal members of the ABC family were first found in the human genome [23], and, subsequently, in all animals [24]. In insects, including *Bombyx mori*, midgut ABC transporters play a role in uric acid metabolism and resistance to insecticides [25,26,27,28], and studies have shown that the ABC transporter specific to the silkworm midgut is related to the response of silkworm molting hormone responses (20-E) [29]. In silkworms of the same sex, the midgut metabolites in individuals fed on an AD and ML were enriched in the ABC transporter signaling pathway and no differences between the sexes were observed, while the intestinal metabolites of silkworms fed on the same diet showed no ABC transporter enrichment (Supplementary Figure S4). This indicates that ADs may affect the antibacterial and disease resistance responses of silkworms. This may be caused by ADs reducing the diversity of their gut bacteria, resulting in a simpler gut microbiota [30]. Even so, our study found that AD-reared silkworms had more midgut differential metabolites than ML-reared silkworms (Table 1). Similarly, previous studies have shown that the number and fold change of various metabolites in the hemolymph of silkworm reared on an AD are higher than those in ML [31]. This may be that an AD is more accessible to ferment than ML, causing intestinal microbial imbalance, and enriching the lactic acid bacteria and other flora in the intestine of silkworms [30], resulting in the diversification of midgut metabolites.

Aminoacyl-tRNA synthetase, also known as aminoacyl-tRNA ligase or amino acid activator, is an enzyme that attaches appropriate amino acids to their tRNA during protein translation and is a key enzyme in protein synthesis. It was found that a large number of metabolites were enriched in the aminoacyl-TRNA synthesis pathway under different dietary regimes, indicating that AD feeding affected protein synthesis in silkworms, resulting in protein differences that could affect silkworm growth, development, and silk yield.

The sex of a silkworm significantly affects its cocoon traits [32], and nutrient utilization and silk synthesis differ greatly between male and female silkworms and affect cocoon size and silk quality [21,32]. Male silkworms have higher economic value than females because they produce smaller cocoons with better silk quality [33,34,35]. For the silk proteins, Gly, alanine (Ala), serine (Ser), and tyrosine (Tyr) account for more than 85% of the total amino acids [12,36,37]. The silk proteins of *B. mori* are mainly composed of sericin and silk fibroin [38,39], and amino acids such as Gly and Ser play an important role in silk fibroin synthesis [40]. In this study, a great deal of differential metabolite enrichment in the Gly, Ser, and threonine (Thr) metabolic pathways was detected in the midgut of male silkworms fed on ML and AD, but not in female silkworms. However, during the long-term rearing of silkworms, we did not observe the difference in feeding behavior between different sexes. In the process of food selection, the silkworm has formed a specific relationship with mulberry over a long period of evolution, and its chemoreceptors play an important role in the feeding process. While olfactory neurons recognize volatile cues, gustatory neurons sense soluble chemicals by contact [41]. BmGrs is a taste receptor gene in silkworms, and several of them show sexual orientation, so that they may be involved in stage-specific or sex-specific behaviors, such as food selection in the larval stage [42]. This may lead to different responses of silkworms of different sexes to diet, leading to differential changes in silk quality and the amount of silk fibroin in the silk protein, suggesting future avenues in the study of sex in silkworms.

Methionine sulfoxide is a free amino acid with two isomeric forms, L(+) and D(+) and is a metabolite of amino acids and their derivatives. Studies have shown that in silkworms, the content and configuration of methionine sulfoxide are related to the instar stage. The concentration of methionine sulfoxide is very low in 5th instar larvae, but increases rapidly at the wandering, pupal, and adult stages. L(+) isomers of methionine sulfoxide have been found in extracts of 1st and 5th instar larvae, adults, and eggs, but not in feces and urine, while D(+) isomers have only been found in pupal extracts [43,44]. The specific timing of expression of methionine sulfoxide indicates that it is related to the onset of diapause [43] and the concentration of L (+)-Met (O) in the hemolymph of diapause silkworms is higher than in non-diapause silkworms [45]. In our study, methionine sulfoxide was the most significantly upregulated metabolite in the MG_AD vs MG_ML and FG_AD vs FG_ML groups, while L-methionine sulfoxide was also the differential metabolite detected in all four diet groups. This metabolite reflects the growth and development status of silkworms. It may be that the nutritional difference between AD and ML leads to an increase in methionine sulfoxide content in the midgut of silkworm, blocking the glutamate synthesis pathway [46,47], thus inhibiting a silkworm’s development and slowing its growth. As a result, silkworms fed on AD grew more slowly than those fed on ML, and the diapause of silkworm eggs may also be promoted.

In order to solve the problem of reduced immunity of silkworms caused by AD in the sericulture industry, some studies have shown the effectiveness of adding beneficial flora to the diet. Feeding with *Lactococcus lactis* and peptidoglycans derived from *L. plantarum* or *L. paraplantarum* 11-1 can increase a silkworm’s resistance to immune tolerant *Pseudomonas aeruginosa* [48,49,50]. Adding phototrophic bacterial to AD can improve disease resistance and the economic value of silkworms [51], and varying the amount added to AD can modify the diet to meet the demands of production. Adding Gly, Ser and other amino acids essential for silk protein synthesis to the diet can increase silk yield. One of the most easily oxidized amino acids is methionine, which can be oxidized to methionine sulfoxide [52]. and reversed using methionine sulfoxide reductase (Msr) [53]. Methionine can regulate the metabolism and anti-oxidation in organisms. The Msr content of silkworms can be increased by changing some of the components of AD, so as to reduce the methionine sulfoxide content.

## Figures and Tables

**Table 1:** Results of the analysis of differential metabolites.

Compared Samples	Number of Total Identify	Number of Total Significant	Number of Significant Up	Number of Significant Down
MGmt_AD. vs. MGmt_ML	758	280	183	97
FGmt_AD. vs. FGmt_ML	758	318	245	73
FGmt_AD. vs. MGmt_AD	758	139	90	49
FGmt_ML. vs. MGmt_ML	758	95	65	30

**Table 2:** Differential metabolites in the midgut of male and female silkworms fed on AD vs. ML.

Metabolites	Class	FC (Female)	FC (Male)
Methionine sulfoxide	Amino acids and their derivatives	11.18	59.43
Nε-(1-carboxymethyl)-L-lysine	Amino acids and their derivatives	27.35	38.38
Lysophosphatidylcholine 16:1	Phospholipid	8.22	12.20
2,6-Diaminooimelic acid	Amino acids and their derivatives	10.66	11.34
Imatinib	Other	7.70	11.32
Sachydrine	Amino acids and their derivatives	8.24	9.11
Lysophosphatidylcholine 15:1	Phospholipid	7.90	6.70
18-Hydroxycorticosterone	Steroids and their derivatives	0.06	0.09
20-Carboxy-Leukotriene B4	Fatty acyl	0.13	0.15
Carnitine-C5	Carnitines	0.17	0.15
2-Methylbutyroylcarnitine	Fatty acyl	0.16	0.16

**Table 3:** Pathway enrichment statistics of differential proteins and differential metabolites in female silkworms.

Map Title	Map ID	Differential Proteins *p* Value	Differential Metabolites *p* Value
Pyruvate metabolism	map00620	1.00	0.75
Drug metabolism–cytochrome P450	map00982	0.22	0.40
Pyrimidine metabolism	map00240	0.15	0.82
Tryptophan metabolism	map00380	0.40	0.53
Valine, leucine, and isoleucine degradation	map00280	0.28	0.22
Glycerolipid metabolism	map00561	0.50	1.00
Lysine degradation	map00310	0.34	0.03
Ascorbate and aldarate metabolism	map00053	0.40	0.12
Histidine metabolism	map00340	0.34	0.78
Glycine, serine, and threonine metabolism	map00260	1.00	0.47
Cysteine and methionine metabolism	map00270	0.49	0.47

**Table 4:** Pathway enrichment statistics of differential proteins and differential metabolites in male silkworms.

Map Title	Map ID	Differential Proteins *p* Value	Differential Metabolites *p* Value
mTOR signaling pathway	map04150	0.24	0.54
Glycerolipid metabolism	map00561	0.40	0.54
Glutathione metabolism	map00480	0.16	1.00
Caffeine metabolism	map00232	0.24	0.10
Arginine biosynthesis	map00220	1.00	0.02
Inositol phosphate metabolism	map00562	0.42	0.66
Phenylalanine metabolism	map00360	0.24	1.00
Drug metabolism–other enzymes	map00983	0.24	0.54
Biosynthesis of amino acids	map01230	0.33	0.02
Thiamine metabolism	map00730	0.42	0.28
Sphingolipid metabolism	map00600	0.06	0.10
Metabolic pathways	map01100	0.63	0.83
Galactose metabolism	map00052	0.15	1.00

## Data Availability

The raw data supporting the conclusions of this article will be made available by the corresponding author, without undue reservation.

## Supplementary Materials

The following are available online at https://www.mdpi.com/article/10.3390/insects14040347/s1, Table S1: Differential metabolites of each experimental group, Table S2: Differential proteins of AD and ML in silkworm midgut, Figure S1: Phenotype observation of *Bombyx mori* in AD group and ML group, Figure S2: QC sample TIC overlay plot, Figure S3: PLS-DA-obtained scatter plot and sorting verification plot, Figure S4: KEGG enrichment of FG_AD vs. MG_AD and FG_ML vs. MG_ML.
