# Supplementary material for: Metabonomic Analysis of Silkworm Midgut Reveals Differences between the Physiological Effects of an Artificial and Mulberry Leaf Diet
Source: Insects. 2023 Mar 31;14(4):347. doi: 10.3390/insects14040347 (PMC10146990; doi:10.3390/insects14040347)
Supplement: Supplementary file 1 [file insects-14-00347-s001.zip › insects-2284645-supplementary.pdf]

Supplementary Table S1: Differential Metabolites of Each Experimental Group.

Compared Samples	Differential Metabolite Name	Retention Time(Min)	Significant up. down
FGmt_AD.vs.FGmt_ ML	20-Carboxy-Leukotriene B4	13.82	down
	2,6-Diaminooimelic Acid	0.94	up
	L-Homocitrulline	0.972	up
	Nε-(1-Carboxymethyl)-L-lysine	0.84	up
	Arachidic acid	12.43	up
	Hexadecanedioate	11.86	up
	Imatinib	11.76	up
	Lysopc 16:1	11.75	up
	N6-Acetyl-L-lysine	1.304	up
	Pantothenic acid	4.57	up
	Calcium D-Panthotenate	4.59	up
	L-Alanine	0.86	up
	LysoPC 15:1	11.45	up
	Xanthosine	3.928	up
	Sarcosine	0.9	up
	2-Methylbutyroylcarnitine	5.45	down
	Carnitine-C5	5.43	down
	Glyceraldehyde 3-phosphate diethyl acetal	0.96	down
	4-Acetamidobutanoate	1.03	up
	N-acetyl-glutamate	1.67	up
	cis-4-Hydroxy-D-proline	2.26	up
	Ethambutol	0.81	down
	9(S)-HPODE	10.83	up
	Vitamin B2	5.94	down
	Stearic acid	2.27	up
	Punicic Acid	12.7	up
	Adipamide	0.84	up
	Alpha-Linolenic acid	12.64	up
	Quinic acid	0.998	down
	D-Proline	0.967	up
	Maleic Acid	0.94	up
	5beta-Androstan-17beta-ol-3-one	0.84	up
	Caffeylalcohol	2.28	up
	5-Hydroxytryptophan	3.165	up
	18-Hydroxycorticosterone	13.52	down
	9,10-Dihome	11.33	up
	(R)-3-Hydroxyisobutyric acid	2.37	down
	Digitoxigenine	6.12	down
	gamma-Glutamylglutamic acid	1.05	up
	Stachydrine	0.97	up
	L-Leucine	2.32	up

N-Acetylalanine	2.15	up
beta-Hydroxyisobutyrate	2.4	down
L-Pyroglutamic acid	1.689	up
Aminomalonic acid	0.88	down
5-oxoproline	1.63	up
N-Oleoyl Glycine	14.62	up
L-allo-Isoleucine	2.28	up
DL-Leucine	2.3	up
Galactaric acid	0.9	up
Urocanic acid	1.04	down
Tricarballylic acid	1.9	up
2-Oxoadipic acid	8.05	up
L-Ornithine	0.762	up
cis-7-Hexadecenoic Acid	11.65	up
Methionine sulfoxide	0.92	up
Kynurenic acid	5.296	down
12,13-EODE	12.67	up
10-Formyl-Thf	4.85	up
geranyl pp	0.83	up
D-Glucarate	0.82	up
Gentisic acid	5.399	up
Ergothioneine	1.03	up
L-2-Aminoadipic acid	0.91	up
Isonicotinic acid	1.49	up
beta-Cubebene	13.54	down
DL-Serine	0.83	up
17alpha-Ethinyl estradiol	13.58	up
13-HPODE	11.45	up
L-Lysine	0.74	up
L-Serine	0.846	up
Orotidine	1	up
N-Acetyl-Dl-glutamic acid	1.84	up
L-Threonine	0.888	down
5-Aminolevulinate	2.07	up
Erucic acid	2.37	down
trans-3-Hydroxy-L-proline	2.14	up
Propionylcholine	1.03	up
D-Lactic acid	1.44	up
Nε,Nε,Nε-trimethyllysine	0.76	down
N-Acetyl-L-glutamic acid	0.71	down
2-Pyrrolidinone	2.04	up
Vitamin U	0.75	down
N6-Succinyl Adenosine	4.54	up
N-Acetylthreonine	1.74	up

6-Aminohexanoate	2.09	up
Nicotinic Acid	1.478	up
Piperidine	2.04	up
L-Saccharopine	1.03	up
L-Citrulline	0.89	up
Lactobionic acid	0.91	up
5-Methoxyindole-3-acetic acid	4.87	up
Methionine	1.46	up
XMP	1.43	up
isoleucine	2.11	up
Picolinic acid	1.48	up
Tiglic acid	1.07	down
Asp-Phe methyl ester	5.36	up
alpha-Cadinene	13.52	down
Trimethyllysine	1.03	down
2-Methylsuccinic Acid	0.94	up
DL-Citrulline	0.9	up
N7-Methylguanosine	4.15	down
Uracil	1.47	up
Lactic acid	1.44	up
Linolelaidic Acid (C18:2N6T)	15.16	up
2-Hydroxy-6-Aminopurine	2.76	up
carbamaoyl asparatate	1.07	down
2-Hydroxy-3-methylbutanoic acid	4.932	down
2,6-Dimethylaniline	4.07	up
Linoleic acid	15.19	up
Lysopa 18:0	13.78	down
L-Glutamic acid monosodium salt	0.86	up
Oleoylcarnitine	11.9	up
gamma-Butyrolactone	2.16	up
Ureidosuccinic acid	1.031	down
Benzamidine Hydrochloride Hydrate	4.09	up
Purine	4.1	up
L-Glutamate	0.886	up
Deoxyribose 5-Phosphate	0.82	up
3-(2-Hydroxyphenyl)propionic acid	4.06	up
7-Methylguanine	4.09	up
O-Aceyl-L-Serine	1.056	up
Guanine	2.72	up
2-(Formylamino)Benzoic Acid	4.07	up
Phthalate	4.07	up
Taurine	0.865	up
dUMP	0.92	up
Lysopc 20:4	12.9	up

Hydroxyglutaric acid	1.45	up
D-Myo-inositol 4,5-bisphosohate	0.97	up
gamma-Nonanolactone	4.1	up
6-Phospho-D-gluconate	0.98	up
D-Phenylalanine	4.106	up
Phloretate	4.05	up
2-Phenylglycine	2.78	up
Dl-Tropic acid	4.09	up
trans-Cinnamic acid	4.07	up
L-Tyrosinemethylester	4.5	up
6-Methylmercaptopurine	4.04	up
Delta-Tridecalactone	9.17	up
DL-Norvaline	1.068	up
Lysopc 18:1	12.9	up
N',N'',N'''-P-Coumaroyl-Cinnamoyl-Caffeoyl Spermidine	4.94	up
Lysopc 16:2 (2N Isomer)	11.13	up
Cuminaldehyde	4.06	up
Methyl beta-D-galactopyranoside	1.03	up
4-Hydroxyisoleucine	1.038	up
NADH	2.79	up
Cis-4,7,10,13,16,19-Docosahexaenoic Acid(C22:6N3)	14.89	up
1-Oleoyl-Sn-Glycero-3-Phosphocholine	12.82	up
Sedoheptulose 7-phosphate	0.87	up
p-Mentha-1,3,8-triene	11.37	up
Xanthurenic Acid	4.977	up
1-Methylxanthine	4.09	down
Oleoyl-L-alpha-lysophosphatidic acid	13.2	up
LysoPE 18:0	13.73	down
N-Methyl-L-arginine hydrochloride	0.95	down
allantoate	1.06	down
5-Hydroxylysine	0.72	up
Methyl alpha-D-glucopyranoside	1.02	up
Pimelic acid	5.77	up
dCMP	1.04	up
3-Hydroxy-butyryl carnitine	1.91	up
Lysine Butyrate	0.9	up
4-Chloro-6-methoxy-2-(methylsulfinyl)pyrimidine	2.72	up
4-Pyridoxic acid	2.94	up
5,6-Dihydro-5-methyluracil	0.69	up
L-Lactic acid	1.487	up
L-Tryptophan	4.894	up

2-Phenylbutyric acid	1.83	up
11-cis-Retinol	12.17	up
Dl-Indole-3-lactic acid	4.87	up
Urea	0.929	up
Homovanillic acid	6.091	down
3-Methylindole	4.87	up
3-Methyladipic acid	5.747	up
L-Tyrosine	1.84	up
L-(-)-alpha-Amino-epsilon-Caprolactam	0.7	up
N-Methyl-a-aminoisobutyric acid	1.22	up
L-Methionine sulfone	1.84	up
6-Deoxy-D-glucose	1.825	up
L-Lysinamide	4.91	up
Hypoxanthine	1.509	down
2-Hydroxy-2-methylbutanedioic acid	1.5	up
D-Homocysteine	1.86	up
L-Aspartic acid	0.86	up
L-(-)-Glyceric acid	1	up
Ribitol	0.927	up
D-Glucuronic acid	0.894	up
Furfural	0.87	up
Lysope 16:0	12.48	down
2-Hydroxycinnamate	1.81	up
Isoquinoline	4.9	up
Deoxyadenosine	2.33	up
Nicotinate ribonucleoside	1.04	down
Methyl nicotinate	5.678	up
2'-Deoxyguanosine-5'-monophosphate	1.66	up
Homocysteic acid	1.83	up
PAF C-16	13.8	down
TPP	0.915	down
1-(4-Methoxyphenyl)-2-propanone	1.81	up
S-Adenosyl-L-methionine	0.8	down
Porphobilinogen	4.9	up
2-(3,4-dimethoxyphenyl)ethanamine	1.81	up
DL-Norepinephrine	0.917	up
Cytidine-5'-monophosphate	0.97	up
N-Acetyl-L-histidine	1	down
LysoPC 18:0	13.8	down
1-Stearoyl-Sn-Glycerol-3-Phosphocholine	13.78	down
dTMP	2.14	up
2-Methylpentanedioic acid	5.012	up
1,5-Anhydro-D-Glucitol	1.11	up
2,3,4-Trihydroxybenzoic acid	4.569	down

N-carbamoyl-L-aspartate	0.9	down
Pyridoxamine	1	down
6-Aminonicotinamide	1.95	up
L-Valine	1.2	up
Methylguanidine HCl	0.88	down
S-ribosyl-L-homocysteine	0.91	up
O-Desmethylnaproxen	11.86	up
UDP-D-glucose	0.95	up
gamma-Muurolene	4.87	up
Lactitol	0.9	up
3-Methylxanthine	1.83	up
L-Erythrulose	1.04	up
9,10-EODE	1.69	up
DL-o-Tyrosine	1.81	up
dAMP	1.47	up
N-P-Coumaroyl Spermidine	3.13	up
Methyl Benzoate	8.39	up
2-Isopropylmalate	5.46	down
Pipecolinic Acid	0.85	up
L-Pipecolate	1.049	up
UDP-galactose	0.92	up
N-Methylnicotinamine	1.82	up
(S)-2-Aminobutanoate	0.947	up
N-Acetylglycine	0.9	up
Guanosine	2.772	up
Cycloleucine	0.84	up
Mesterolone	11.586	down
L-Hydroxylysine	0.9	up
UMP	1.037	up
D-2-Aminobutyric acid	0.947	up
Val-Ser	1.01	down
Itaconic acid	1.45	up
S-adenosyl-L-methioninamine	0.86	down
Dl-Norleucine	0.9	up
N,N-Dimethylglycine	0.9	up
Phe-Phe	6.51	up
UDP-D-glucuronate	0.94	up
Flucytosine	1	up
Methylmalonate	1.938	up
N-(3-Indolylacetyl)-L-Alanine	3.97	up
Taurodeoxycholic acid sodium salt	10.504	down
Aspartic acid di-O-glucoside	0.97	up
6 phosphod gluconate	1.05	up
1-Aminocyclopropane-1-carboxylic acid	0.89	up

N-Acetyl-Asp-Glu	2.02	up
Gamma-Glu-Leu	5.09	up
Betaine	0.907	up
Asp-glu	0.96	up
Citraconic acid	1.56	up
Ethylmalonate	6.37	up
Gly-Tyr	3.09	down
L-Homoserine	1.9	up
Mevalonic acid	2.06	up
Succinic acid	1.936	up
8-Bromoguanosine	0.98	up
alpha-Ketoglutaric acid	1.18	up
Glycochenodeoxycholic acid	10.48	down
N-Acetyl-L-leucine	1.04	up
L-Gulono-1,4-lactone	0.948	up
Choline Chloride	0.83	up
Glycohyocholic acid Sodium salt	8.98	up
Lysopc 15:0	12.93	up
Imidazoleacetic acid	0.91	up
Cytidine 3'-phosphate	1.22	up
Pipecolic acid	1.042	up
Choline Hydroxide	0.828	up
Sulfoacetic acid	0.95	down
3-Ketodihydrosphingosine	11.13	down
Sedoheptulose 1,7-bisphosphate	0.88	up
dGMP	1.04	up
Uric Acid	1.14	down
3-Aminoisobutyric acid	0.931	up
Ala-gly	0.84	up
DL-3,4-Dihydroxyphenyl glycol	7.37	up
Noradrenaline	6.36	up
3-Hydroxy-hexadecanoyl carnitine	10.82	down
D-Ribose 5-phophate	0.89	up
Isoursodeoxycholic acid	11.93	up
L-Homocystine	0.83	up
N alpha-Acetyl-L-Arginine	1.03	down
Ectoine	0.95	down
3-Hydroxy-3-Methylpentane-1,5-Dioic Acid	0.87	down
Cadaverine	0.706	up
Hexadecenoylcarnitine	11.52	down
Acetylchloline Chloride	0.9	down
L-Carnitine	0.87	down
N-Acetylhistamine	2.73	up

	1-Palmitoyl-Sn-Glycero-3-Phosphocholine	12.52	down
	Adipic Acid	0.84	up
	Guggulsterone	15.17	down
	dCDP	1.02	up
	Xylitol	0.92	up
	O-Acetyl-L-homoserine	1	down
	4-Nitrophenol	7.71	down
	Lysopa 16:0	12.54	down
	Oxaceprol	1.5	down
	2,3-Dihydroxybenzoic acid	5.963	down
	1,2,3-Benzenetricarboxylic acid	6.42	up
	3b,7b-Dihydroxy-5-androsten-17-one	0.96	up
	ADP-ribose	1.05	up
	alpha-Nicotinamide adenine dinucleotide	1.04	down
	N-Acetyl-L-methionine	5.315	up
	L-Arabinose	0.9	down
	2-deoxyglucose-6-phosphate	0.98	down
FGmt_AD.vs.MGmt_AD	D-Glucose 6-phosphate	0.92	down
	D-Fructose 6-phosphate	0.87	down
	D-Mannose 6-phosphate	0.87	down
	N-Oleoyl Glycine	14.62	up
	N,N-Dimethylglycine	0.9	up
	Dodecanoylcarnitine	10	up
	Choline Hydroxide	0.828	up
	Octadeca-11E,13E,15Z-trienoic acid	13.5	up
	Choline Chloride	0.83	up
	alpha-D-Galactose 1-phosphate	0.87	down
	Xanthosine	3.928	up
	2-Phosphoglyceric acid	0.92	up
	Carnitine-C12	9.94	up
	Pyridoxine O-Glucoside	0.99	down
	6-Phospho-D-glucono-1,5-lactone	0.97	down
	L-Cystathionine	0.84	up
	D-2-Aminobutyric acid	0.947	up
	(S)-2-Aminobutanoate	0.947	up
	gamma-Glutamylglutamic acid	1.05	up
	N-Acetylalanine	2.15	up
	Phthalic Anhydride	13.53	up
	L-Tyrosinemethylester	4.5	up
	Linolelaidic Acid (C18:2N6T)	15.16	up
	Linoleic acid	15.19	up
	Adenylocuccinic Acid	3.68	up
	XMP	1.43	up
	3-Aminoisobutyric acid	0.931	up

Tetradecanoylcarnitine	10.88	up
2,4-Dihydroxybenzoic Acid	1.7	up
S-Adenosyl-L-methionine	0.8	down
Carnitine-C14	10.83	up
Isonicotinamide	1.51	up
Azelaic acid	7.433	down
Carbamoylphosphate	0.86	up
Mag (18:1)	15.23	down
Succinic anhydride	1.02	down
Guggulsterone	15.17	down
Lysope 16:0	12.48	down
Stearoylcarnitine	12.41	up
Nicotinamide	1.539	up
Pyrophosphate	0.97	down
D-Glucosamine 6-phosphate	0.86	up
Xanthine	1.714	up
5-Aminosalicylate	1.68	up
Ectoine	0.95	down
Carnitine-C18	12.4	up
Picolinamide	1.51	up
L-Dihydroorotic Acid	1.051	up
S-Sulfo-L-cysteine	0.92	up
S-adenosyl-L-methioninamine	0.86	down
dTMP	2.14	up
N-(5-Aminopentyl)acetamide	1.01	down
Lysope 14:0	11.27	down
ARCAR	1.06	down
2-C-Methyl-D-erythritol 2,4-cyclodiphosphate	0.89	down
L-Glutamic acid monosodium salt	0.86	up
TPP	0.915	up
Deoxyribose 5-Phosphate	0.82	up
MAG (18:2)	14.64	down
beta-Nicotinamide adenine dinucleotide phosphate	1.03	up
dCMP	1.04	up
L-Saccharopine	1.03	up
Oxypurinol	1.68	up
O-Aceyl-L-Serine	1.056	up
1,3-diphopshateglycerate	12	down
1-Palmitoyl-Sn-Glycero-3-Phosphocholine	12.52	down
Glycerol-3-phosphate	0.9	up
1-Methylguanosine	4.04	up
13,14-dihydro-15-keto-PGD2	1.04	up

Hexanoylcarnitine	6.6	up
Trehalose 6-phosphate	0.96	down
Thiamine	0.81	down
geranyl pp	0.83	up
LysoPE 18:0	13.73	down
1,4-Dihydro-1-Methyl-4-Oxo-3-Pyridinecarboxamide	1.69	up
L-Pyroglutamic acid	1.689	up
4-Hydroxyisoleucine	1.038	up
Uracil	1.47	up
1,4-Naphthoquinone	1.082	down
Deoxycytidine	1.81	up
1-Methylguanine	4.03	up
Acetylcholine	1.02	down
5-oxoproline	1.63	up
O-Phospho-L-serine	0.83	up
5-Hydroxytryptophol	1.03	down
L-Leucyl-L-alanine Hydrate	3	up
3-Amino-4-methylpentanoic acid	1.862	down
beta-Alanine methyl ester	0.98	down
2-Phenylbutyric acid	1.83	up
1,3 bisphopshoglycerate	12.04	down
3b,7b-Dihydroxy-5-androsten-17-one	0.96	up
dUMP	0.92	up
3-Hydroxy-hexadecanoyl carnitine	10.82	up
Ethyl myristate	15.68	up
5alpha-Pregnane-3,20-dione	1.86	down
Mevalonic acid	2.06	up
4-aminobutyrate	0.84	down
Biopterin	2.097	down
2-Furoic acid	4.832	down
Cystine	0.84	up
glutathione disulfide	1.81	up
DL-o-Tyrosine	1.81	up
Cyclohexylsulfamate	1.2	down
Phe-Phe	6.51	up
L-Cysteine-glutathione gisulfide	0.96	up
Oxidized glutathione	1.78	up
2'-Deoxyguanosine-5'-monophosphate	1.66	up
4-Aminobutyric acid	0.864	down
alpha-Ketoglutaric acid	1.18	up
Lysope 18:1	12.81	down
Phosphopyruvic acid	1.03	up
Lysopg 18:1	13.81	down

	Hexadecenoylcarnitine	11.52	down
	Decanoylcarnitine	9.04	up
	PE(18:1(9Z)/0:0)	12.85	down
	Glutathione	1.51	down
	L-Homocystine	0.83	up
	3-OH-anthranilate	1.68	up
	Prostaglandin B2	0.7	up
	Cytidine-5-Triphosphate	1.79	up
	Choline Glycerophosphate	0.89	up
	N',N'',N'''-P-Coumaroyl-Cinnamoyl-Caffeoyl Spermidine	4.94	up
	4-Methylpentanoate	0.89	down
	Irbesartan	1.04	up
	Tauro-alpha-Muricholic acid sodium salt	9.01	down
	R-Aminobutyrate	0.91	down
	5,6-Dihydro-5-methyluracil	0.69	up
	L-(-)-alpha-Amino-epsilon-Caprolactam	0.7	up
	Hexadecanoic acid	15.7	up
	1-(4-Methoxyphenyl)-2-propanone	1.81	up
	Spermine	0.73	up
	L-Methionine sulfone	1.84	up
	D-Fructose 1,6-bisphosphate	0.86	down
	N-Formylkynurenine	4.2	down
	Asp-glu	0.96	up
	Lysopa 16:0	12.54	down
	D-Homocysteine	1.86	up
	L-Ascorbate	1.03	up
	Lysopa 18:0	13.78	down
FGmt_ML.vs.MGmt_ML	Benzoic Acid	7.42	up
	N7-Methylguanosine	4.15	up
	D-Glucuronic acid	0.894	down
	L-Cystathionine	0.84	up
	Methyl beta-D-galactopyranoside	1.03	down
	Hydrocortisone 17-butyrate	10.51	down
	Vitamin B2	5.94	up
	Homovanillic acid	6.091	up
	Methyl alpha-D-glucopyranoside	1.02	down
	2,3,4-Trihydroxybenzoic acid	4.569	up
	8-Hydroxyguanosine	3.09	up
	1-Methylxanthine	4.09	up
	Deoxyguanosine	3.181	up
	Methionine sulfoxide	0.92	up
	1-Methylguanosine	4.04	up
	Digitoxigenine	6.12	up

1-Methylguanine	4.03	up
Nelarabine	4.11	up
2-(Dimethylamino)Guanosine	4.57	up
N4-Acetylcytidine	4.38	up
3-Methylsalicylate	0.71	down
Dl-3-Hydroxynorvaline	0.85	up
O-Desmethylnaproxen	11.86	down
N6-Succinyl Adenosine	4.54	down
2,3-Dihydroxybenzoic acid	5.963	up
6-Deoxy-D-glucose	1.825	up
NADH	2.79	down
N-acetyl-glutamate	1.67	down
Xanthosine	3.928	up
N-Acetyl-D-glucosamine 6-phosphate	1.81	up
D-Homocysteine	1.86	up
N-Acetyl-Dl-glutamic acid	1.84	down
1-(4-Methoxyphenyl)-2-propanone	1.81	up
3-Hydroxypropanoic acid	1.15	down
Trans,Trans-Muconic Acid	0.96	down
2,4-Dihydroxybenzoic Acid	1.7	down
L-arginino-succinate	0.84	up
L-Tyrosine	1.84	up
S-Adenosyl-L-methionine	0.8	up
Allantoin	0.943	up
Azelaic acid	7.433	up
Oxaceprol	1.5	up
6-Aminonicotinamide	1.95	up
p-Mentha-1,3,8-triene	11.37	down
LysoPC 10:0	6.25	up
Thymidine	4.289	down
Styrene	10.53	down
1-Palmitoyl-Sn-Glycero-3-Phosphocholine	12.52	up
L-Rhamnose monohydrate	0.98	up
L-Methionine sulfone	1.84	up
Homocysteic acid	1.83	up
Lysopc 17:0	13.17	up
Lysopg 18:1	13.81	up
Hexadecenoylcarnitine	11.52	up
2-Hydroxycinnamate	1.81	up
alpha-Cadinene	13.52	up
2-Phenylbutyric acid	1.83	up
2-Furoic acid	4.832	up
PE(18:1(9Z)/0:0)	12.85	up
Erythronolactone	4.03	down

	L-Fucose	0.98	up
	Kynurenic acid	5.296	up
	Lactic acid	1.44	down
	Lysope 18:1	12.81	up
	Pantetheine	5.84	down
	3-Hydroxyisovalerate	4.084	down
	D-Lactic acid	1.44	down
	Lysopa 18:0	13.78	up
	LysoPC 20:2	13.26	up
	Tyramine	1.98	up
	Lysope 14:0	11.27	up
	Deoxycytidine	1.81	up
	Lysopc 16:0	12.57	up
	5-oxoproline	1.63	down
	beta-Cubebene	13.54	up
	Melamine	0.9	up
	Linoleoylcarnitine	11.41	up
	1-Caffeoylquinic Acid	18.71	down
	L-Pyroglutamic acid	1.689	down
	L-Palmitoylcarnitine	11.69	up
	Isonicotinic acid	1.49	down
	Fexofenadine	12.04	up
	Lysopc 18:3	12.57	up
	(R)-Lipoic acid	9.4	down
	L-Lactic acid	1.487	down
	DL-o-Tyrosine	1.81	up
	3-Ketodihydrosphingosine	11.13	up
	Lysopc 15:0	12.93	up
	Stearoylcarnitine	12.41	up
	Carnitine-C16	11.65	up
	Diethylglutarate	0.67	down
	Hydroxypyruvic acid	2.17	down
	Nicotinic Acid	1.478	down
	LysoPE 18:2	12.08	up
	Carnitine-C18	12.4	up
MGmt_AD.vs.MGmt_ML	Nε-(1-Carboxymethyl)-L-lysine	0.84	up
	Stachydrine	0.97	up
	Methionine sulfoxide	0.92	up
	Lysopc 18:1	12.9	up
	1-Oleoyl-Sn-Glycero-3-Phosphocholine	12.82	up
	Glyceraldehyde 3-phosphate diethyl acetal	0.96	down
	Lysopc 20:4	12.9	up
	Imatinib	11.76	up
	Lysopc 16:1	11.75	up

L-Homocitrulline	0.972	up
18-Hydroxycorticosterone	13.52	down
2-Hydroxy-3-methylbutanoic acid	4.932	down
Octadeca-11E,13E,15Z-trienoic acid	13.5	down
Phthalic Anhydride	13.53	down
20-Carboxy-Leukotriene B4	13.82	down
Cis-4,7,10,13,16,19-Docosahexaenoic Acid(C22:6N3)	14.89	up
2,6-Diaminooimelic Acid	0.94	up
Oleoyl-L-alpha-lysophosphatidic acid	13.2	up
8-Hydroxyguanosine	3.09	up
Propionylcholine	1.03	up
Arachidic acid	12.43	up
Erucic acid	2.37	down
2-Methylsuccinic Acid	0.94	up
L-Alanine	0.86	up
Fexofenadine	12.04	up
D-Mannose 6-phosphate	0.87	up
D-Glucose 6-phosphate	0.92	up
Ergothioneine	1.03	up
cis-7-Hexadecenoic Acid	11.65	up
3-Hydroxy-butyryl carnitine	1.91	up
Sarcosine	0.9	up
D-Fructose 6-phosphate	0.87	up
Lysopc 15:0	12.93	up
alpha-D-Galactose 1-phosphate	0.87	up
Thymine	7.29	up
2-Methylbutyroylcarnitine	5.45	down
Carnitine-C5	5.43	down
Taurine	0.865	up
Nonadecanoic acid	18.28	down
2,4-Dihydroxybenzoic Acid	1.7	down
3-Hydroxy-hexadecanoyl carnitine	10.82	down
LysoPC 15:1	11.45	up
L-Leucyl-L-alanine Hydrate	3	down
Azelaic acid	7.433	up
11-cis-Retinol	12.17	up
Carnitine-C14	10.83	down
L-Serine	0.846	up
Ethambutol	0.81	down
Hypoxanthine	1.509	down
Oleoylcarnitine	11.9	up
Xanthine	1.714	down
Adipamide	0.84	up

Anthranilic acid	1.44	down
Oxypurinol	1.68	down
carbamaoyl asparatate	1.07	down
Delta-Tridecalactone	9.17	up
Ureidosuccinic acid	1.031	down
TPP	0.915	down
1,4-Dihydro-1-Methyl-4-Oxo-3-Pyridinecarboxamide	1.69	down
Xanthosine	3.928	up
allantoate	1.06	down
PE(18:1(9Z)/0:0)	12.85	up
5-Hydroxytryptophol	1.03	up
Lysopc 18:2	12.16	up
Lysope 18:1	12.81	up
Sulfoacetic acid	0.95	down
Quinic acid	0.998	down
(R)-Lipoic acid	9.4	down
6-Phospho-D-glucono-1,5-lactone	0.97	up
Tetradecanoylcarnitine	10.88	down
MAG (18:2)	14.64	up
Dodecanoylcarnitine	10	down
Carnitine-C12	9.94	down
Lysopc 17:0	13.17	up
5beta-Androstan-17beta-ol-3-one	0.84	up
5-Hydroxytryptophan	3.165	up
Isoursodeoxycholic acid	11.93	up
2-Methylpentanedioic acid	5.012	up
Aminomalonic acid	0.88	down
L-Erythrulose	1.04	up
Nicotinate ribonucleoside	1.04	down
gamma-Glutamylglutamic acid	1.05	up
N-Methyl-L-arginine hydrochloride	0.95	down
N-Acetyl-D-glucosamine 6-phosphate	1.81	up
Calcium D-Panthotenate	4.59	up
Hexadecanedioate	11.86	up
5-Aminosalicylate	1.68	down
L-Threonine	0.888	down
Lactitol	0.9	up
Creatine phosphate disodium salt	0.93	down
13-HPODE	11.45	up
3-OH-anthranilate	1.68	down
13,14-dihydro-15-keto-PGD2	1.04	down
Val-Ser	1.01	down
Calcium phosphorylcholine chloride	0.87	down

Thiamine	0.81	up
Nε,Nε,Nε-trimethyllysine	0.76	down
Glu-Glu	1	down
Beta-Alanine	4.54	up
L-Aspartic acid	0.86	up
L-Ornithine	0.762	up
Taurodeoxycholic acid sodium salt	10.504	down
L-Ascorbate	1.03	down
Hydrocortisone 17-butyrate	10.51	down
D-Myo-inositol 4-monophosohate	0.92	up
2-Oxoadipic acid	8.05	up
Pantothenic acid	4.57	up
N-carbamoyl-L-aspartate	0.9	down
Lysope 14:0	11.27	up
17alpha-Ethinyl estradiol	13.58	up
Tricarballylic acid	1.9	up
4-Acetamidobutanoate	1.03	up
Orotidine	1	up
N-Acetyl-L-glutamic acid	0.71	down
Trimethyllysine	1.03	down
Lysopg 18:1	13.81	up
5-Methyl-dl-tryptophan	4.26	down
Punicic Acid	12.7	up
Alpha-Linolenic acid	12.64	up
Vitamin U	0.75	down
LysoPE 18:2	12.08	up
Urocanic acid	1.04	down
beta-Hydroxyisobutyrate	2.4	down
(R)-3-Hydroxyisobutyric acid	2.37	down
Acetylchloline Chloride	0.9	down
Pyridoxine O-Glucoside	0.99	up
Mag (18:1)	15.23	up
D-Fructose 1,6-bisphosphate	0.86	up
Lysops 22:6	13.14	up
N-P-Coumaroyl Spermidine	3.13	up
Guanosine monophosphate	1.01	up
Acetylcholine	1.02	up
L-Citrulline	0.89	up
N-Acetyl-L-leucine	1.04	up
S-ribosyl-L-homocysteine	0.91	up
N6-Acetyl-L-lysine	1.304	up
Uric Acid	1.14	down
D-Myo-inositol 4,5-bisphosohate	0.97	up
Thymidine 3,5-cyclic monophosphate	4.2	down

12,13-EODE	12.67	up
DL-Citrulline	0.9	up
Asp-Phe methyl ester	5.36	up
2-Aminooctanoic acid	1.03	up
Lactobionic acid	0.91	up
beta-Cubebene	13.54	down
2-Furoic acid	4.832	up
L-Hydroxylysine	0.9	up
N-Formylkynurenine	4.2	up
LysoPC 20:2	13.26	up
(+,-)-Octopamine HCl	1.034	down
Carbamoylphosphate	0.86	down
L-Histidinol	0.95	down
Gly-Tyr	3.09	down
Lysopc 16:2 (2N Isomer)	11.13	up
Deoxyadenosine	2.33	up
L-Arabinose	0.9	down
5-Hydroxylysine	0.72	up
2-Isopropylmalate	5.46	down
DL-Serine	0.83	up
Methylguanidine HCl	0.88	down
alpha-Cadinene	13.52	down
Methionine	1.46	up
10-Formyl-Thf	4.85	up
Carnitine-C3	2.56	down
Dl-3-Hydroxynorvaline	0.85	up
Propionyl-L-carnitine	2.57	down
4-Chloro-6-methoxy-2-(methylsulfinyl)pyrimidine	2.72	up
Lysopc 14:0	11.37	up
Methylmalonate	1.938	up
Tolfenamic acid	2.15	down
4-Pyridoxic acid	2.94	up
L-Alanyl-L-Lysine	2.57	down
Phosphocreatine	0.91	down
13-Hpotre(R)	11.93	up
L-arginino-succinate	0.84	up
Imidazoleacetic acid	0.91	up
1,3-diphopshateglycerate	12	up
Isonicotinamide	1.51	down
L-Lysine	0.74	up
Succinic acid	1.936	up
1,3 bisphopshoglycerate	12.04	up
Nicotinamide	1.539	down

Urea	0.929	up
Nandrolone	9.45	up
L-allo-Isoleucine	2.28	up
Kynurenic acid	5.296	down
beta-Nicotinamide mononucleotide	0.993	up
Gly-Phe	4.88	down
6-Deoxy-D-glucose	1.825	up
methylamino-l-alanine	1.74	up
Pyrophosphate	0.97	up
Cyclohexylsulfamate	1.2	up
UDP-galactose	0.92	up
Adenylocuccinic Acid	3.68	down
D-Lactic acid	1.44	up
Picolinamide	1.51	down
8-Bromoguanosine	0.98	up
2-Hydroxy-6-Aminopurine	2.76	up
L-(-)-Glyceric acid	1	up
NG,NG-Dimethyl-L-arginine	0.93	down
Trehalose 6-phosphate	0.96	up
7-Ketocholesterol	16.92	up
2-deoxyglucose-6-phosphate	0.98	down
UDP-D-glucose	0.95	up
CTP	0.87	up
(5-L-Glutamyl)-L-Amino Acid	1.18	up
Ala-Gln	0.85	up
aminoimidazole carboxamide ribonucleotide	16.98	up
gamma-Butyrolactone	2.16	up
L-Tyrosine	1.84	up
L-Leucine	2.32	up
2-Phenylglycine	2.78	up
DL-Leucine	2.3	up
D-Erythrose 4-phosphate	0.95	up
2-Phenylbutyric acid	1.83	up
Melamine	0.9	up
Lysine Butyrate	0.9	up
Spermine	0.73	down
2-Phosphoglyceric acid	0.92	down
1-(4-Methoxyphenyl)-2-propanone	1.81	up
L-Methionine sulfone	1.84	up
O-Phospho-L-serine	0.83	down
6-Aminonicotinamide	1.95	up
3-Methylsalicylate	0.71	down
D-Proline	0.967	up

Dopamine HCl	1.5	down
Gentisic acid	5.399	up
2-Hydroxycinnamate	1.81	up
Hexanoylcarnitine	6.6	down
Glycine	0.88	down
3-O-Feruloylquinic acid	18.11	up
Homoarginine	0.807	up
Biotin	6.003	down
3-Methylxanthine	1.83	up
Lactic acid	1.44	up
Lysopc 20:0	15.1	up
L-2-Aminoadipic acid	0.91	up
Phosphoethanolamine	0.86	down
Guanidinoacetic acid	1.13	down
N-Acetyl-5-hydroxytryptamine	2.63	down
N-lactoyl-phenylalanine	6.97	up
D-Homocysteine	1.86	up
L-Arginine	0.794	up
Maleic Acid	0.94	up
N-Acetylglycine	0.9	up
Allitol	0.91	down
Trimethylamine N-oxide	0.872	down
Betaine	0.907	up
Homocysteic acid	1.83	up
cGMP	1.98	up
2-Pyrrolidinone	2.04	up
Sorbitol	0.91	down
scyllo-Inositol	0.853	up
N-Acetyl-L-tyrosine	5.17	up
trans-3-Hydroxy-L-proline	2.14	up
Indole-3-acetamide	0.81	up
L-Lactic acid	1.487	up
Piperidine	2.04	up
Glycerol-3-phosphate	0.9	down
5-Hydroxymethyluracil	1.041	up
Dl-Norleucine	0.9	up
Caffeylalcohol	2.28	up
isoleucine	2.11	up
alpha-D-Glucose	0.912	up
Phthalate	4.07	up
Stearic acid	2.27	up
Histamine	0.699	down
Tiglic acid	1.07	down
cis-4-Hydroxy-D-proline	2.26	up

beta-Nicotinamide adenine dinucleotide phosphate	1.03	down
2-(Formylamino)Benzoic Acid	4.07	up
7-Methylguanine	4.09	up
5-Aminolevulinate	2.07	up
Benzamidine Hydrochloride Hydrate	4.09	up
Phloretate	4.05	up
Purine	4.1	up
6-Methylmercaptopurine	4.04	up
6-Aminohexanoate	2.09	up
1-Caffeoylquinic Acid	18.71	down
D-Phenylalanine	4.106	up

Supplementary Table S2: Differential proteins of AD and ML in silkworm midgut.

Silkworm sex	Protein	AD.vs.ML Up . Down	Interpro_Term
Female	P_KWMTBOMO10647	up	Serine proteases, trypsin domain
	P_KWMTBOMO16346	up	NodB homology domain
	P_KWMTBOMO02956	up	Serine proteases, trypsin domain
	P_KWMTBOMO00254	down	Triosephosphate isomerase
	P_KWMTBOMO09265	up	Haemolymph juvenile hormone binding
	P_KWMTBOMO11900	up	Lepidopteran low molecular weight lipoprotein
	P_KWMTBOMO06428	up	Aminotransferase class-III
	P_KWMTBOMO09263	up	Haemolymph juvenile hormone binding
	P_KWMTBOMO13101	up	--
	P_KWMTBOMO03769	up	Serine proteases, trypsin domain
	P_KWMTBOMO13182	up	Glycoside hydrolase family 16
	P_KWMTBOMO01029	up	Serine proteases, trypsin domain
	P_KWMTBOMO06105	down	Gelsolin-like domain
	P_KWMTBOMO12672	down	Fructose-1-6-bisphosphatase class I, N-terminal
	P_KWMTBOMO07611	up	Carboxylesterase, type B
	P_KWMTBOMO08820	down	Nucleoside diphosphate kinase
	P_KWMTBOMO12719	up	Serine proteases, trypsin domain
	P_KWMTBOMO09266	up	Haemolymph juvenile hormone binding
	P_KWMTBOMO14447	down	Glycoside hydrolase family 31; Glycoside hydrolase family 31, N-terminal domain; Domain of unknown function DUF5110
	P_KWMTBOMO14257	up	Lepidopteran low molecular weight lipoprotein
	P_KWMTBOMO12723	up	Serine proteases, trypsin domain
	P_KWMTBOMO02278	down	Translationally controlled tumour protein
	P_KWMTBOMO09429	up	Serine proteases, trypsin domain
	P_KWMTBOMO03670	down	Glutathione S-transferase, N-terminal; Glutathione S-transferase, C-terminal
	P_KWMTBOMO09466	up	Serine proteases, trypsin domain
	P_KWMTBOMO04899	up	Peptidase M1, membrane alanine aminopeptidase, N-terminal
	P_KWMTBOMO16179	down	Serpin domain
	P_KWMTBOMO03216	down	FKBP-type peptidyl-prolyl cis-trans isomerase domain; Tetratricopeptide repeat 2
	P_KWMTBOMO07145	up	Aldehyde dehydrogenase domain
	P_KWMTBOMO04283	down	Reverse transcriptase domain
	P_KWMTBOMO04887	up	Peptidase M1, membrane alanine aminopeptidase, N-terminal

	P_KWMTBOMO10746	down	MD-2-related lipid-recognition domain
	P_KWMTBOMO16345	up	NodB homology domain
	P_KWMTBOMO06451	up	Beta-1,3-glucan-binding protein, N-terminal
	P_KWMTBOMO07102	up	Glycoside hydrolase family 1
	P_KWMTBOMO15086	up	Peptidase M14, carboxypeptidase A
	P_KWMTBOMO15034	up	Lipocalin/cytosolic fatty-acid binding domain
	P_KWMTBOMO15118	up	Transcriptional coactivator/pterin dehydratase
	P_KWMTBOMO12304	up	Band 4.1, C-terminal
	P_KWMTBOMO11963	up	Reverse transcriptase domain; Endonuclease/exonuclease/phosphatase
	P_KWMTBOMO09031	up	Glycerophosphodiester phosphodiesterase domain
	P_KWMTBOMO02998	up	Integrase, catalytic core; Tumour necrosis factor domain; Reverse transcriptase, RNA-dependent DNA polymerase; GAG-pre-integrase domain
	P_KWMTBOMO14416	down	Mu homology domain
	P_KWMTBOMO11115	up	MD-2-related lipid-recognition domain
Male	P_KWMTBOMO08926	down	Serpin domain
	P_KWMTBOMO15337	down	Thrombospondin type-1 (TSP1) repeat; Pancreatic trypsin inhibitor Kunitz domain; ADAM-TS Spacer 1; PLAC; Immunoglobulin I-set
	P_KWMTBOMO00254	down	Triosephosphate isomerase
	P_KWMTBOMO07918	up	DNA/RNA non-specific endonuclease
	P_KWMTBOMO06239	down	Peptidase M17, leucyl aminopeptidase, C-terminal
	P_KWMTBOMO09265	up	Haemolymph juvenile hormone binding
	P_KWMTBOMO08642	down	Heat shock protein 70 family
	P_KWMTBOMO06428	down	Aminotransferase class-III
	P_KWMTBOMO09263	up	Haemolymph juvenile hormone binding
	P_KWMTBOMO08280	up	Serine proteases, trypsin domain
	P_KWMTBOMO10407	down	Peptidase T2, asparaginase 2
	P_KWMTBOMO06056	down	Domain of unknown function DUF1907
	P_KWMTBOMO13182	up	Glycoside hydrolase family 16
	P_KWMTBOMO04900	up	Peptidase M1, membrane alanine aminopeptidase, N-terminal; ERAP1-like C-terminal domain
	P_KWMTBOMO01131	up	Translation elongation factor, IF5A C-terminal
	P_KWMTBOMO13370	down	Signal recognition particle, SRP72 subunit, RNA-binding; Putative TPR-like repeat

P_KWMTBOMO08184	up	Peptidase M14, carboxypeptidase A; Carboxypeptidase, activation peptide
P_KWMTBOMO08445	down	Sushi/SCR/CCP domain; von Willebrand factor, type D domain; NIDO domain; AMOP domain
P_KWMTBOMO13530	down	Superoxide dismutase, copper/zinc binding domain
P_KWMTBOMO01843	down	Peptidase S9, prolyl oligopeptidase, catalytic domain; Dipeptidylpeptidase IV, N-terminal domain
P_KWMTBOMO00984	up	NADP-dependent oxidoreductase domain
P_KWMTBOMO12672	down	Fructose-1-6-bisphosphatase class I, N-terminal
P_KWMTBOMO07611	up	Carboxylesterase, type B
P_KWMTBOMO08820	down	Nucleoside diphosphate kinase
P_KWMTBOMO00995	up	NADP-dependent oxidoreductase domain
P_KWMTBOMO09598	down	NADH:ubiquinone oxidoreductase subunit B14.5a
P_KWMTBOMO09266	up	Haemolymph juvenile hormone binding
P_KWMTBOMO14447	down	Glycoside hydrolase family 31; Glycoside hydrolase family 31, N-terminal domain; Domain of unknown function DUF5110
P_KWMTBOMO13483	up	Lepidopteran low molecular weight lipoprotein
P_KWMTBOMO07821	down	Transaldolase/Fructose-6-phosphate aldolase
P_KWMTBOMO12723	up	Serine proteases, trypsin domain
P_KWMTBOMO12073	down	Reverse transcriptase domain; Transcription activator MBF2
P_KWMTBOMO02278	down	Translationally controlled tumour protein
P_KWMTBOMO09429	up	Serine proteases, trypsin domain
P_KWMTBOMO07282	up	Partial AB-hydrolase lipase domain
P_KWMTBOMO16270	up	Lipid transport protein, N-terminal; Lipid transport, open beta-sheet; Vitellinogen, open beta-sheet
P_KWMTBOMO02269	down	Ubiquinol cytochrome reductase, transmembrane domain; Ubiquinol-cytochrome c reductase 8kDa, N-terminal; Rieske [2Fe-2S] iron-sulphur domain
P_KWMTBOMO13171	up	RNA recognition motif domain; Eukaryotic translation initiation factor 3 subunit G, N-terminal
P_KWMTBOMO04889	up	ERAP1-like C-terminal domain
P_KWMTBOMO05535	down	Aldehyde dehydrogenase domain

P_KWMTBOMO09466	up	Serine proteases, trypsin domain
P_KWMTBOMO11064	down	Heat shock protein 70 family
P_KWMTBOMO07701	down	Peptidase S9, prolyl oligopeptidase, catalytic domain; Dipeptidylpeptidase IV, N-terminal domain
P_KWMTBOMO01315	down	ATPase, V1 complex, subunit H, C-terminal
P_KWMTBOMO02587	up	Lipase/vitellogenin
P_KWMTBOMO01785	down	Protein kinase domain
P_KWMTBOMO13443	up	Trypsin Inhibitor-like, cysteine rich domain
P_KWMTBOMO00317	down	--
P_KWMTBOMO00094	up	Laminin IV; Reverse transcriptase domain; Integrase, catalytic core; Laminin EGF domain; Laminin, N-terminal
P_KWMTBOMO15985	up	EGF-like calcium-binding domain; Complement Clr-like EGF domain
P_KWMTBOMO04899	up	Peptidase M1, membrane alanine aminopeptidase, N-terminal
P_KWMTBOMO04892	up	Peptidase M1, membrane alanine aminopeptidase, N-terminal; ERAP1-like C-terminal domain
P_KWMTBOMO01581	up	EF-hand domain
P_KWMTBOMO16179	down	Serpin domain
P_KWMTBOMO02958	up	Serine proteases, trypsin domain
P_KWMTBOMO08204	up	Arf GTPase activating protein
P_KWMTBOMO16273	up	Glycoside hydrolase, family 22
P_KWMTBOMO16447	up	Aminoglycoside phosphotransferase
P_KWMTBOMO03216	down	FKBP-type peptidyl-prolyl cis-trans isomerase domain; Tetratricopeptide repeat 2
P_KWMTBOMO06227	down	RNA-binding protein Lupus La
P_KWMTBOMO09262	up	--
P_KWMTBOMO07498	up	LDLR class B repeat; NIDO domain; G2 nidogen/fibulin G2F; EGF domain
P_KWMTBOMO05865	down	Short-chain dehydrogenase/reductase SDR
P_KWMTBOMO04283	down	Reverse transcriptase domain
P_KWMTBOMO05729	up	Beta-galactosidase jelly roll domain; Glycoside hydrolase 35, catalytic domain
P_KWMTBOMO12483	down	Ribosomal protein S19/S15
P_KWMTBOMO06467	down	--
P_KWMTBOMO11008	down	Calponin homology domain; Spectrin repeat; EF-hand, Ca insensitive
P_KWMTBOMO03450	up	PDZ domain
P_KWMTBOMO02529	down	Adenylate kinase, active site lid domain
P_KWMTBOMO03625	up	Alpha/beta hydrolase fold-1

P_KWMTBOMO08056	up	GDP dissociation inhibitor
P_KWMTBOMO10746	down	MD-2-related lipid-recognition domain
P_KWMTBOMO16345	up	NodB homology domain
P_KWMTBOMO08003	down	Endonuclease/exonuclease/phosphatase
P_KWMTBOMO15675	down	5-formyltetrahydrofolate cyclo-ligase
P_KWMTBOMO14599	up	Ferritin/DPS protein domain
P_KWMTBOMO01376	up	Integrin beta subunit, VWA domain; EGF-like domain, extracellular; Integrin beta subunit, cytoplasmic domain
P_KWMTBOMO13991	down	Coproporphyrinogen III oxidase, aerobic
P_KWMTBOMO15715	down	--
P_KWMTBOMO04587	up	NUDIX hydrolase domain
P_KWMTBOMO09274	up	Aminotransferase, class I/classII
P_KWMTBOMO10187	up	Alcohol dehydrogenase, N-terminal
P_KWMTBOMO09714	down	Calcineurin-like phosphoesterase domain, ApaH type
P_KWMTBOMO10542	down	Acyl-CoA oxidase/dehydrogenase, central domain; Acyl-CoA dehydrogenase/oxidase C-terminal; Acyl-CoA dehydrogenase/oxidase, N-terminal
P_KWMTBOMO05258	up	Peptidase M24
P_KWMTBOMO15086	up	Peptidase M14, carboxypeptidase A
P_KWMTBOMO12611	up	Ubiquitin domain; Ubiquitin-associated domain
P_KWMTBOMO08760	up	Phosphoglycerate kinase
P_KWMTBOMO03083	up	Zinc finger, UBP-type; Histone deacetylase domain
P_KWMTBOMO08648	up	SH3 domain; Spectrin repeat; EF-hand domain; EF-hand, Ca insensitive
P_KWMTBOMO07215	down	Aldehyde oxidase/xanthine dehydrogenase, a/b hammerhead; 2Fe-2S ferredoxin-type iron-sulfur binding domain; Molybdopterin dehydrogenase, FAD-binding; [2Fe-2S]-binding; CO dehydrogenase flavoprotein, C-terminal; Aldehyde oxidase/xanthine dehydrogenase, molybdopterin binding
P_KWMTBOMO07378	up	Serpin domain
P_KWMTBOMO01972	down	PITH domain
P_KWMTBOMO09712	up	5'-Nucleotidase, C-terminal
P_KWMTBOMO09674	down	Macrophage migration inhibitory factor
P_KWMTBOMO05541	down	Cation-transporting P-type ATPase, N-terminal; Cation-transporting P-type ATPase, C-terminal; P-type ATPase, A domain; HAD-like domain

P_KWMTBOMO16674	up	--
P_KWMTBOMO07952	up	WHEP-TRS domain; Anticodon-binding
P_KWMTBOMO06180	up	--
P_KWMTBOMO03245	up	SWAP/Surp; Ubiquitin domain; Splicing factor 3A subunit 1
P_KWMTBOMO09054	up	Isopropylmalate dehydrogenase-like domain
P_KWMTBOMO14416	down	Mu homology domain
P_KWMTBOMO16000	up	Calponin homology domain; EF-hand domain

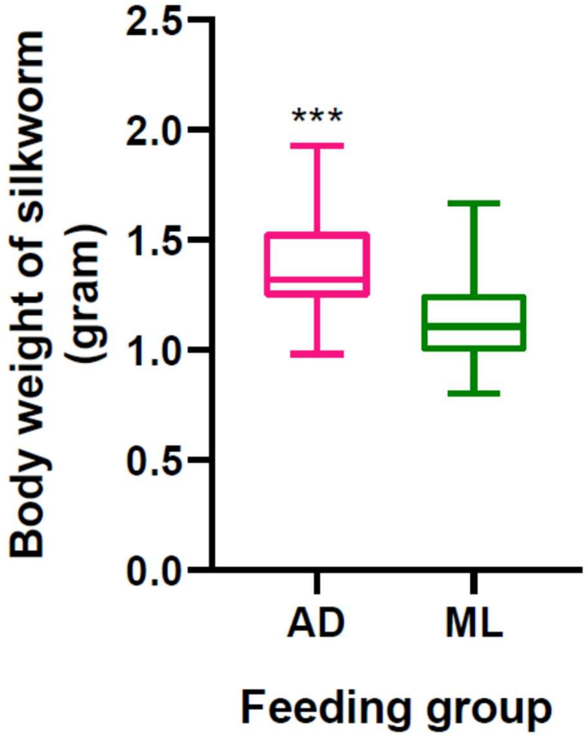

Supplementary Figure S1. Phenotype observation and body-weight statistics of silkworm fed with fresh mulberry leaves (ML) and artificial diet (AD). Fifty silkworm larvae on day 3 of the fifth instar were randomly selected from the AD group and the ML group, respectively, and measured the body weight. AD, artificial diets; ML, mulberry leaves. The asterisks indicates a difference in significance (*** p-value < 0.001).

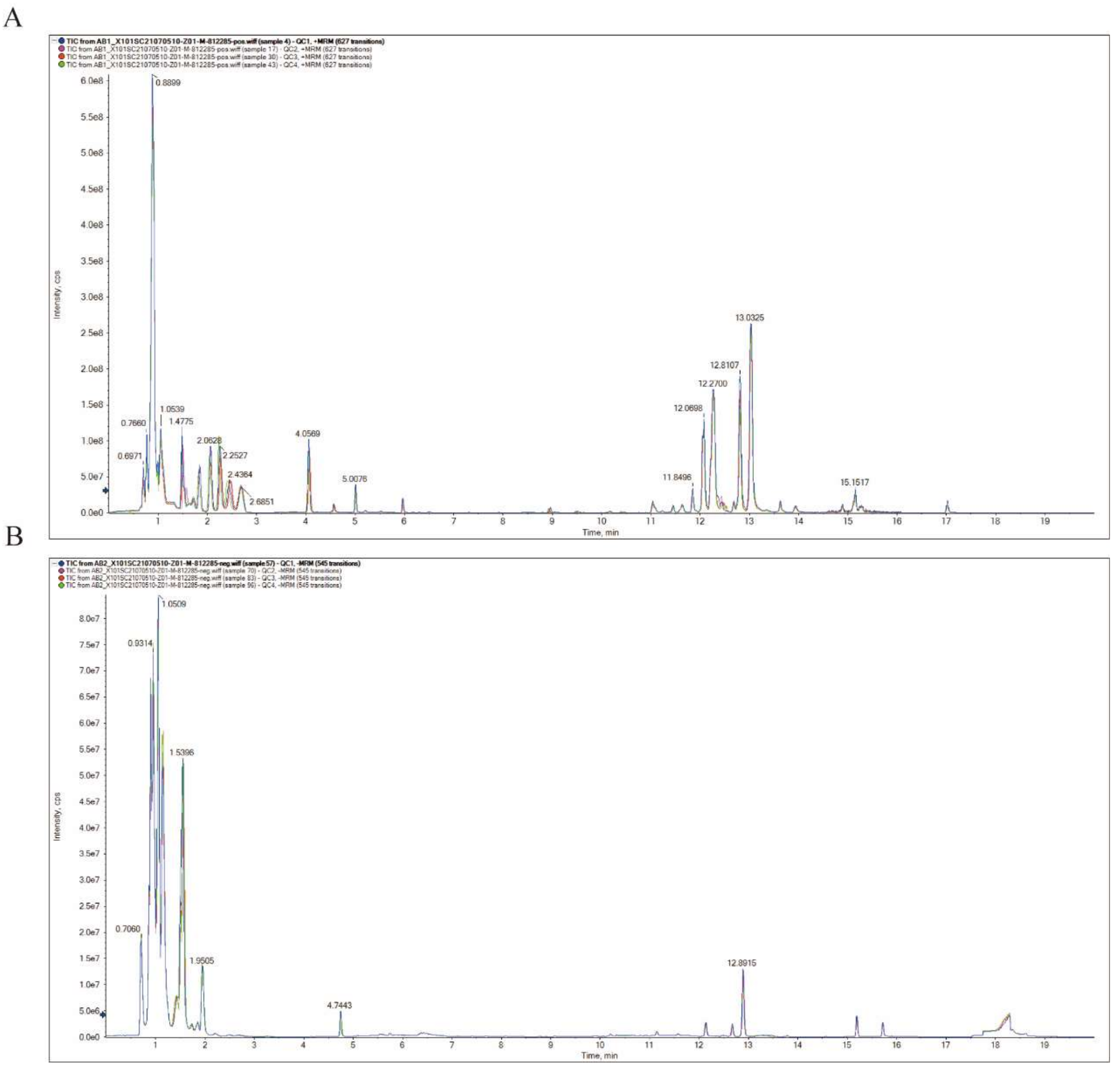

Supplementary Figure S2. (A) QC sample TIC overlay plot (positive ion mode plot); (B) QC sample TIC overlay plot (negative ion mode plot).

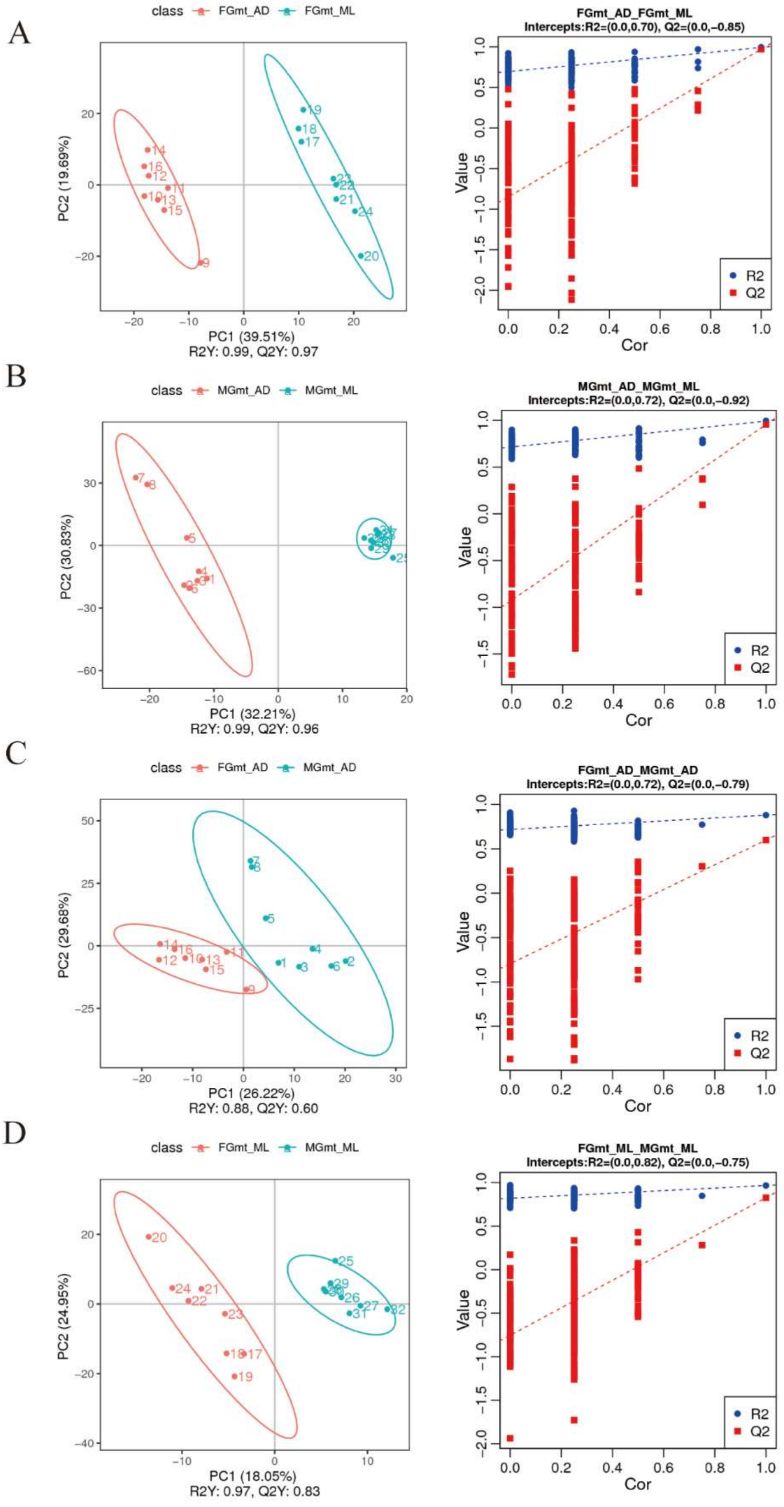

Supplementary Figure S3. PLS-DA obtained scatter plot and sorting verification plot. (A) FG_AD vs FG_ML; (B) MG_AD vs MG_ML; (C) FG_AD vs MG_AD; (D) FG_ML vs MG_ML. Scatter plot according to PLS-DA we can see that the R2Y and Q2Y values in the four sets of experiments are all close to 1, thus indicating that the mode type a is more stable and reliable. The value of R2 is larger than that of Q2 and the intercept between Q2 regression line and Y axis is less than 0, which shows that we built the established model is not "over-fitted", which can describe the experimental samples better and the construction of the model is standard indeed.

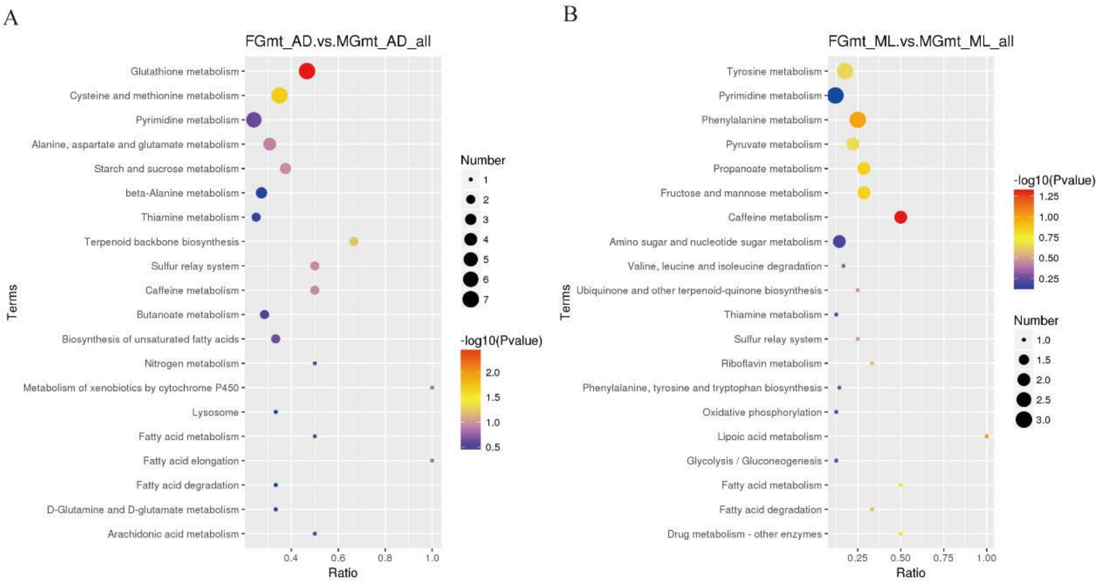

Supplementary Figure S4. KEGG enrichment. (A) FG_AD vs MG_AD; (B) FG_ML vs MG_ML. Only the top 20 differential metabolite enrichment pathways are shown. The horizontal coordinate is x/y (the number of differential metabolites in the corresponding metabolic pathway/the number of total metabolites identified in that pathway), with larger values indicating greater enrichment of differential metabolites in that pathway. The colour of the dot represents the p-value of the hypergeometric test, the smaller the value, the more reliable and statistically significant the test is. The size of the dot represents the number of differential metabolites in the corresponding pathway; the larger the dot, the more differential metabolites are present within that pathway.
